# Multidimensional analysis of conventional and electronic cigarette consumption among students

**DOI:** 10.3389/fpsyg.2025.1724372

**Published:** 2026-01-30

**Authors:** Carmen Maria Țîru, Claudiu Coman, Costel Marian Dalban, Vlad Bătrânu-Pințea, Mihail Anton, Vasile Gherheș, Daniel-Rareș Obadă, Adrian Otovescu

**Affiliations:** 1 Department for Teacher Training, West University, Timișoara, Romania; 2 Academy of Romanian Scientists, Brașov, Romania; 3 Department of Social Sciences and Communication, Transilvania University, Brașov, Romania; 4 Department of Sociology and Social Work, Alexandru Ioan Cuza University, Iași, Romania; 5 National Defense University Carol I, Bucharest, Romania; 6 Department of Communication and Foreign Languages, The Polytechnic University, Timișoara, Romania; 7 Department of Communication Sciences and Public Relations, Alexandru Ioan Cuza University, Iași, Romania; 8 Department of Communication, Journalism and Educational Sciences, Craiova University, Craiova, Romania

**Keywords:** e-cigarettes, psychosocial factors, public health, smoking behavior, students

## Abstract

**Introduction:**

Cigarette consumption among university students remains a complex public health issue, encompassing both conventional and electronic products. This study investigates the multidimensional aspects of cigarette use among young adults, with a particular focus on psychosocial, perceptual, and behavioral determinants influencing nicotine consumption.

**Methods:**

A quantitative, cross-sectional design was employed. Data were collected through a standardized online questionnaire administered to 267 students enrolled in higher education institutions in Brașov, Romania. Descriptive statistics, correlation analyses, and ANOVA were used to identify consumption patterns, differentiate user types (current smokers, former smokers, and non-smokers), and examine emotional and motivational factors associated with tobacco and e-cigarette use.

**Results:**

Findings indicate that traditional cigarette smoking is most frequently initiated under social influence, whereas electronic cigarette use is primarily associated with aesthetic appeal and flavor variety. Psychological motivations, particularly stress relief and social comfort, show significant correlations with continued nicotine use, especially among current smokers. Analysis informed by the Theory of Planned Behavior and Social Cognitive Theory highlights the role of behavioral intention and self-efficacy in sustaining smoking behaviors.

**Discussion:**

The results underscore the multifaceted nature of smoking behavior among youth populations, revealing a dynamic interplay between cognitive, emotional, and environmental variables. These findings provide empirical support for the development of targeted intervention strategies and emphasize the need for integrated public health policies that address both product regulation and psychosocial education.

## 1 Introduction

The main objective of this research is to analyze students’ smoking behaviors in a comprehensive manner, with respect to the use of both conventional and electronic cigarettes, including patterns of use and associated factors (Purba et al., 2022; Dharmarajlu et al., 2024).

The central research question concerns the prevalence of exclusive conventional cigarette use, exclusive electronic cigarette use, as well as dual use within the studied sample, while also examining how social, psychological, and demographic variables correlate with these patterns of use.

Both the aim and the research question fall within a broader body of studies and debates in the field. Tobacco use continues to be one of the leading preventable causes of mortality worldwide, generating over eight million deaths annually, of which approximately 1.3 million are attributable to exposure to secondhand smoke (Altet et al., 2022). The use of electronic cigarettes has increased noticeably among adolescents and young adults worldwide, including among those with no prior smoking experience (Atuegwu et al., 2021). For example, in England, the prevalence of regular vaping among adults increased from approximately 1% in 2013 to about 10% in 2023, with the sharpest rise among younger people (Jackson et al., 2024). Numerous countries have also noted an increase in the experimentation with and use of electronic cigarettes on university campuses. Studies from the Middle East report an e-cigarette use prevalence of 14% among university students in Qatar (with no significant gender differences) and around 21–24% among students in Saudi Arabia, underscoring the global scale of this phenomenon (Kurdi et al., 2021; Alzahrani et al., 2023). These figures contrast with adult populations, where e-cigarette use is generally lower, suggesting that university-aged young people constitute a high-risk group for adopting new tobacco products (Rocha-Ávila et al., 2025).

The theoretical framework of this research is grounded in the Theory of Planned Behavior (TPB), according to which an individual’s intention to adopt a given behavior represents a central determinant of that behavior (Ajzen, 1991). Complementarily, Social Cognitive Theory (SCT) underscores the role of self-efficacy and observational learning in shaping human conduct (Bandura, 1982). Both perspectives contribute to understanding smoking behavior, including by highlighting community-level implications (Boyd et al., 2021). At the same time, an additional line of analysis, derived from Consumer Culture Theory, proposes applying the concept of “jouissance” to explain the practice of smoking, which makes it possible to extend traditional approaches to the phenomenon of consumption (Ehnhage, 2024).

The study makes original contributions on four essential fronts. The first dimension consists in operationalizing the trajectories between conventional smoking and e-cigarette use, by introducing variables such as “smoking evolution” and by testing demographic and psychological associations. Second, the research proposes a detailed quantification of the emotional functions associated with smoking and vaping, analyzing how these correlate with an intention to quit. A third aspect concerns integrating into a unified analytical framework the motives specific to e-cigarette use, such as the variety of flavors, aesthetic attractiveness, and the degree of social acceptability in restrictive contexts. Finally, the segmentation of the investigated population explicitly includes the category of dual users, thus offering a multidimensional perspective that contributes to the development of the recent international literature devoted to university populations.

The international literature on smoking and vaping behaviors among students is already substantial, yet several significant gaps persist. A considerable share of existing studies either focuses exclusively on the consumption of conventional cigarettes, examines the use of electronic cigarettes in isolation, or limits itself to investigating only a single dimension of the phenomenon.

In Romania, as well as in Eastern Europe, up-to-date data on the concurrent use of the two types of products among the student population remain insufficient. Although studies have examined smoking among young people in Romania, (Popa et al., 2021), the literature does not provide recent analyses with a multidimensional character that simultaneously integrate traditional smoking and vaping, together with the correlated factors, within a university setting. This gap is all the more relevant as cultural and regulatory differences can directly influence these behaviors. In this context, the present study seeks to address this need through a comprehensive analysis of conventional and electronic cigarette use among students at a leading public university in Brașov, Romania.

## 2 Theoretical analyses in relation to cigarette consumption

### 2.1 Smoking behavior

In recent years, the consumption of cigarettes—both conventional and electronic—has shown an upward trend among young people, which has raised concerns about the possibility that the use of electronic cigarettes may serve as a facilitator for the transition to conventional smoking (Bold et al., 2017). From a neurological perspective, nicotine acts as a stimulant of the central nervous system, exerting its effects by binding to nicotinic acetylcholine receptors in the brain (Kurdyś-Bykowska et al., 2024). In France, recent data show that 28% of students are smokers, and among these approximately 15% already exhibit a high level of dependence (Pasquereau et al., 2017; Mauduy et al., 2023). Although smoking has declined, it continues to represent a major cause of mortality and of physical and mental disorders (Suhányi et al., 2020). An analysis published in *BMC Public Health* reports a global prevalence of current use of electronic nicotine delivery systems (ENDS) of approximately 10.2% among pupils and students, with a higher value in Europe, around 12.7%, and with sex differences, the frequency being higher among males (Albadrani et al., 2024).

In Turkey, at a medical university, 52.3% of e-cigarette users also reported using conventional cigarettes—indicating dual use—and current e-cigarette use was estimated at approximately 4% (Dilektasli et al., 2024). In the United States, a large nationwide study (n ≈ 332,721 students) found that dual users had significantly higher odds of engaging in binge drinking compared with exclusive cigarette users or exclusive e-cigarette users (Thornquist and Qeadan, 2025).

Regional and global estimates suggest that approximately four million adolescents aged 13–15 use tobacco products in the WHO European Region (WHO Global Report, 2025). With regard to national smoking prevalence, it is estimated at 25.9% in Romania, 31.6% in Bulgaria, and 27.8% in Hungary (WHO GTCR, 2023). Comparative analyses at the level of the European Union show the highest youth smoking rates in Hungary (26%) and Austria (19%), while the lowest values are found in Sweden (5%) and Finland (7%) (Destatis Statistiches Bundesamt, 2021). In the United States, current e-cigarette use among youth has decreased substantially, from 2.13 million (7.7%) in 2023 to 1.63 million (5.9%) in 2024 (FDA Report (U.S. Food and Drug Administration), 2025).

The use of electronic cigarettes is associated with lower nicotine dependence and reduced tobacco consumption compared to conventional smoking. However, existing smoking cessation programs have proven more effective in achieving abstinence, and e-cigarette use did not lead to improvements in quality of life (Rabenstein et al., 2024). In addition, a higher proportion of e-cigarette users reported perceiving their health as good compared to traditional cigarette smokers (Adebisi and Bafail, 2025). Nevertheless, certain risks persist, such as the identification of high concentrations of formaldehyde in the aerosols generated by e-cigarettes, according to a study conducted in Surabaya, Indonesia (Lestari et al., 2018).

Globally, the framework of bans and regulations regarding e-cigarettes and electronic nicotine delivery systems varies considerably across jurisdictions. Tobacco control policies implemented in university settings can contribute to reducing the use of these products and decreasing exposure to ambient secondhand smoke (Dilektasli et al., 2024; Bartington et al., 2020). As shown in literature, it seems that e-cigarette consumption represents lower risk of certain problems, for example stroke is more prevalent in traditional smokers than among e-cigarette users, but, paradoxically, the incidence of stroke was early in onset in e-cigarette smokers compared to traditional smokers (Patel et al., 2022). A second study has shown that e-cigarette vapor can induce an inflammatory response in the respiratory system. However, the levels were significantly higher in conventional smokers (Janik et al., 2025). Finally, e-cigarette use is associated with an elevated, though comparatively lower, cardiovascular risk profile than traditional smoking (Chad, 2025).

### 2.2 Perceptions and attitudes about smoking

In the university setting, the relationship between the level of knowledge, attitudes toward smoking, and actual consumption practices has been a constant object of research (Sabila et al., 2025; Kumar et al., 2024; Alves et al., 2022). The profile of the current smoker indicates a higher likelihood of being young, unmarried, less inclined to perceive smoking as a major health risk, and more frequently residing in environments where other smokers are present, compared with former smokers or those who have never smoked. Social influence factors prove to be important predictors of the onset of tobacco use, and ethnic and racial differences highlight significant variations in prevalence (Mccuistian et al., 2021).

Electronic cigarettes can be interpreted as elements of a modern, health-oriented identity, capable of meeting consumers’ desire for social differentiation (SDS) in relation to traditional smokers. In contrast, conventional cigarettes continue to convey symbolic values linked to elegance or rebellion, thereby shaping social perceptions of smoking (ODS). In a study conducted on a sample of 418 students, the results showed that men and students enrolled in non-health fields were more likely to consider electronic cigarettes less harmful, with perceptions also varying according to the type of device used, as in the case of pod systems compared with other forms of e-cig[arette]. (Vilcassim et al., 2025). From a perspective specific to consumer culture theory, applying the concept of “jouissance” to the practice of smoking offers an expanded interpretive framework, going beyond conventional understandings of this behavior (Ehnhage, 2024).

Some students perceive the consumption of alternative products—such as heated tobacco or electronic systems with and without nicotine—as “safer” and more enjoyable, although these products often contain potentially harmful ingredients, such as artificial flavorings or glycerol (Alma et al., 2022). Tobacco users anticipate a moderate level of difficulty in complying with university policies that mandate a tobacco-free campus (Pignataro and Daramola, 2020). Favorable attitudes toward smoking are found particularly among student smokers, those who have friends who smoke, and individuals exposed to secondhand smoke (SHS) (Alves et al., 2022). At the same time, a segment of students exhibits aggressive behavior toward social attitudes perceived as overly judgmental, and approximately 58% consider smoking or vaping to be “socially acceptable,” although the level of knowledge about university policies regarding on-campus use proves to be uneven (Maqsood et al., 2024).

Aesthetic and functional factors—such as the compact, appealing design of devices or the diversity of flavors—can increase attractiveness and, in turn, intensify use (Thangeswaran et al., 2025; Hoi, 2021). Added to these determinants is the influence exerted by social media in shaping perceptions: it associates e-cigarette use with health-related values and mediates the impact of anti-tobacco online messages, while pro-vaping content tends to reinforce the portrayal of these products as “safer” options (Zhang et al., 2024).

### 2.3 Psychological and emotional motivations

Among the frequently cited psychological and emotional motivations are stress reduction, identity reshaping, and the management of transitional stages (education, employment, leaving the parental home); among these, stress reduction and self-gratification often constitute the principal reasons for smoking (Alsarray et al., 2010). The relationship between smoking and mental health is, however, simultaneous and bidirectional; depressive symptoms, novelty seeking, stress level, and exposure to tobacco advertising are positively correlated with smoking behavior, whereas pro-health behaviors exert a negative influence (Habib et al., 2024; Yang et al., 2022).

In a sample of 55,654 students, 95% of e-cigarette users also reported using other substances (alcohol: 58%; cannabis: 23%). Compared with students reporting no substance use, co-use of e-cigarettes and other substances was associated with higher psychological distress and a recent diagnosis or treatment for a mental health disorder (Kava et al., 2024). Recent data indicate that, among young people who vape, the prevalence of moderate-to-severe depressive and anxiety symptoms is approximately double that of non-users; at the same time, these youths exhibit a higher level of nicotine dependence and report initiation and maintenance motives tied to stress and anxiety (VanFrank et al., 2025).

With regard to the initial motivation for smoking, most students begin under the influence of their circle of friends; having a larger number of smoker friends increases the likelihood of adopting this behavior. Imitation of family members and curiosity also play a significant role, and smoking is frequently used as a stress-management strategy (Ahmed et al., 2020; Hossain et al., 2017; Bin Abdulrahman et al., 2022). For adolescents and young adults, including former smokers, electronic cigarettes may represent an alternative; however, unlike other quit attempts, users do not distance themselves from their social networks, with smoking and e-cigarette use thereby helping to maintain social ties (Wilson et al., 2022).

The development of a recurrent habit and the consolidation of a tradition can contribute to the perpetuation of use; some young people aged 18–35 report feeling physically better while smoking, which favors the emergence of dependence, and the level of risk awareness may be reduced among young health-care professionals (Daama et al., 2023; Mărginean et al., 2025).

Other motives—particularly pharmacological in nature—have also been identified: users consume nicotine to improve mood, either directly or by alleviating withdrawal symptoms, as well as to enhance mental or physical performance (Gherman and Arhiri, 2016).

Based on the literature, it is necessary to further understand smoking behaviors among students and analyze students’ perceptions toward smoking, either conventional or electronic cigarettes. It is of interest to analyze if the increasing trend of choosing electronic cigarettes over conventional ones is still increasing among students, and also to assess what is the level of impact on health while being a smoker is, according to the students. The subject is of interest and relevant, as it has been studied prior in literature as well as in this research, as there is an increasing smoking-related behavior, especially among students and young generations in general.

## 3 Materials and methods

The main purpose of this research is to study the behaviors, perceptions and motivations associated with the consumption of conventional and electronic cigarettes among students. The study aims to identify the differences between current smokers, former smokers and non-smokers, as well as the psychosocial factors that influence the decision to smoke or quit smoking.

In accordance with the stated purpose, the research proposes the following specific objectives:

- O1: Analysis of cigarette consumption behavior among students, with emphasis on the typology of smoking, its duration and the context of initiation.
- O2: Analysis of students’ perceptions and attitudes toward electronic cigarettes, in relation to conventional ones, from the perspective of attractiveness, utility and social acceptability.
- O3: Identification of the main emotional and psychosocial motivations that determine and support smoking behavior among students.
- O4. Identification of patterns of cigarette consumers from the analyzed target audience.

Our research is quantitative, cross-sectional and non-experimental. The instrument used was a standardized questionnaire, applied in online format, consisting exclusively of closed questions. The questionnaire was developed specifically for this study, based on a synthesis of previous research on smoking behaviors, attitudes toward smoking and the use of conventional and electronic cigarettes, and psychosocial motivations for smoking. The questions in sections 12–41 were formulated on a 7-point Likert scale (from “1 – Totally disagree” to “7 – Totally agree”), which allowed the assessment of attitudes and beliefs in a graded manner, and questions 1–11 have nominal answers.

The analyzed sample was represented by students from higher education institutions, within the city of Brasov, selected based on a non-probabilistic sampling method, by convenience. Thus, we distributed the questionnaire in the online environment, through social networks and internal university platforms, and participation was voluntary and anonymous. This sampling strategy is justified by the exploratory nature of the study and the need to access a specific population group—university students—within a limited time frame and with available resources. According to Etikan et al. (2016), convenience sampling is widely used in social sciences when the aim is to identify patterns, trends, or associations in a target group rather than to produce generalizable results. Moreover, this method is considered appropriate when the study focuses on perceptual variables that are stable within relatively homogeneous subgroups, such as students from the same educational and cultural context. Data collection took place during the period 1 March - 30 May, 2025 and obtained the approval of the University of Transilvania Ethics Commission of the Faculty of Sociology and Communication Council, with no. 12 of 23rd of February 2025.

In terms of variables and research questions, we analyzed the Cronbach’s Alpha coefficient, which obtained a coefficient of 0.883 for the scale used, which indicates a high level of internal consistency and suggests that the included items coherently measure the same latent dimension. The set of variables that we used in the analysis was structured into four main dimensions: smoking behavior, attitudes and perceptions toward smoking and the use of electronic cigarettes, awareness of psychological and emotional motivations associated with smoking (Supplementary Appendix Table A1).

We performed the analysis of the above variables using the SPSS (Statistical Package for the Social Sciences) program. To interpret the results, we used descriptive statistics (frequencies, means, standard deviations), difference tests (*t*-test, ANOVA) to compare variables between groups, as well as correlation analyses (Pearson or Spearman, depending on the data distribution). The significance level was set at *p* < 0.05.

Research hypotheses:

*H1*: Students who started smoking with conventional cigarettes are more likely to remain long-term users of this type, compared to those who started with electronic cigarettes.

*H2*: Positive perception of the aesthetics and aroma of electronic cigarettes is associated with an increased likelihood of transitioning from conventional to electronic smoking.

*H3*: Students who smoke to manage negative emotions (anger, anxiety, tension) will exhibit certain behaviors and a reduced intention to quit smoking.

Following the data collection process, we found that the final sample consisted of 267 respondents, all of whom were students enrolled in higher education institutions. Analyzing the data set obtained, it does not present missing values in terms of the socio-demographic variables included in the study, which shows us a higher level of accuracy and robustness of the data, strengthening confidence in the validity of the results.

Analyzing the sample structure from the perspective of geographical origin, we manage to outline a predominantly national profile of the investigated population: 89.9% of the participants are Romanian citizens. The remaining 10.1% are international students coming from various countries, including Mauritius, France, Iran, Italy, Pakistan, Portugal, the Republic of Moldova, Belarus and the United Kingdom. Each of these nationalities are represented in small proportions, without significantly influencing the cultural homogeneity of the group. We note that these students are temporarily in Romania, within international academic mobility programs, such as Erasmus+ or other forms of inter-institutional educational exchanges.

Regarding the gender distribution, we find a female predominance: 70.8% of respondents are women, compared to 28.5% men, and 0.7% chose not to declare their gender. This disproportion may indicate either a real representation of the student population in the sample, or a tendency for women to be more actively involved in social research. Regarding the area of residence, we observed that the majority of respondents come from urban areas (79.4%), while 20.6% are from rural areas. This aspect is coherent with the educational reality, given that university centers are generally located in urban areas, and a large part of students study and live in large cities.

From the distribution of the sample by age group, we can outline a specific profile of the population analyzed. The majority of respondents (79.8%) fall into the 18–25 age group, a category corresponding predominantly to undergraduate students. The representation of other age groups is considerably lower: 9.7% of participants are between 26 and 35 years old, 5.2% between 36 and 45 years old, and people over 45 represent a marginal percentage, below the 6% threshold.

Regarding the educational level achieved, the analyzed data indicates a percentage of 51.7 of respondents who indicated high school graduation as the last completed level, while 47.2% reported completing a higher education level, such as an undergraduate degree or an equivalent program. This structure confirms that most participants belong to the university population, in transition between the pre-university and tertiary levels.

## 4 Results

### 4.1 Smoking behavior

From the analysis of the participants’ smoking behavior, the analyzed data indicate a significant prevalence of tobacco use among the students included in the sample. Thus, we found that 81.6% of the respondents declare themselves active smokers, while only 7.9% do not smoke, and 10.5% are former smokers who have given up this habit. We believe that this distribution highlights a high level of tobacco consumption, with important implications for health and risk behavior among young people. Regarding the duration of smoking, we observed that most of the smoking participants declare that they have smoked for a relatively short time: 43.4% have smoked for a maximum of 3 years, and another 19.9% fall within the range of 3–5 years. Only 18.4% have smoked for 5–8 years, 4.1% between 8 and 10 years, and 14.2% declare that they have smoked for over 10 years. From the interpretation of these data, we appreciate that they can be correlated with the young age of the majority of the sample, suggesting an early initiation, but also a possible continuity of smoking behavior over time.

Regarding the type of cigarette used at the beginning of consumption, we observed that the majority of respondents (80.1%) started with conventional cigarettes, while only 19.9% were initiated by using electronic cigarettes. We consider this aspect important for understanding consumption trajectories and for analyzing how modern alternatives, such as e-cigarettes, can influence the initial behavior of young consumers.

To analyze whether there is a statistically significant association between the type of cigarette with which respondents started smoking and the type of cigarette they currently consume, we used the Chi-square test of independence. The results obtained revealed a statistically significant association between the two variables [χ^2^(3) = 20.288, *p* < 0.001], which suggests that the initial type of cigarette significantly influences current smoking behavior. This association is also confirmed by the symmetric measures: Phi = 0.276 and Cramér’s *V* = 0.276, indicating a medium intensity association between the two nominal variables. The Kendall’s tau-b coefficient = 0.150, with *p* = 0.009, strengthens this link from an ordinal perspective (albeit weak), signaling a positive correlation between the ordinal variables.

Analyzing the data presented in Supplementary Appendix Table A2, we observed that, within the total sample (*N* = 267), 27.0% (*n* = 72) of respondents report having started with conventional cigarettes and continuing to smoke the same type, 19.1% (*n* = 51) report having started with conventional cigarettes but now using only electronic cigarettes, and 23.2% (*n* = 62) report using both conventional and electronic cigarettes. Similarly, 10.1% (*n* = 27) of respondents report having started with electronic cigarettes and continuing to use them, whereas 1.9% (*n* = 5) report switching from electronic to conventional cigarettes and 4.5% (*n* = 12) report combining both types.

We thus find that this mobility between forms of consumption reflects flexible trends in the adoption of smoking-related behaviors, as well as a possible dynamic perception of the risks and benefits of each type of product. To better understand the evolution of behavior from regular cigarettes to electronic cigarettes, we created a new variable “Smoking evolution” (Supplementary Appendix Table A3) built from two other variables Q8 (What type of cigarette did you start with?) and Q9 (What type of cigarette do you smoke now?). The new values of the variable are represented by people who are in the following situations: 1. stayed on conventional, 2. switched to electronic, 3. combine both, 4. quit smoking, 5. stayed on electronic 6. switched from electronic to conventional.

From our analysis, we can highlight that the ANOVA results indicate the existence of statistically significant differences regarding the variable “smoking_evolution” according to demographic factors (such as gender, age and educational level), but especially according to psychological motivations and perceptions associated with smoking. Motives such as relaxation, anxiety reduction, taste pleasure, curiosity or aesthetic perception of electronic cigarettes have proven to be significantly associated with how respondents have evolved in their nicotine consumption behavior.

Thus, by using the ANOVA-Turkey HSD analysis, we identified the following important aspects:

#### 4.1.1 Relationship with age

The 46–55 age group differs significantly from all other age categories in terms of the evolution of smoking behavior, according to the results of the ANOVA test (*F* = 4.171, *p* = 0.001). *Post hoc* analyses highlight significant differences between this group and the younger segments, especially those between 18 and 25 years (mean difference = −1.339, *p* < 0.001), 26–35 years (difference = −1.356, *p* < 0.001) and 36–45 years (difference = −1.314, *p* < 0.001). These results suggest that people aged 46–55 years show increased stability in smoking behavior, being less inclined to adopt alternatives such as electronic cigarettes or to quit completely. In contrast, younger respondents, especially those under 35, demonstrate greater flexibility, being more likely to experiment with alternative types of smoking or even quit.

#### 4.1.2 Relationship with duration of smoking

We found that the duration of smoking experience significantly influences the evolution of consumption habits, according to the results of the ANOVA analysis (*F* = 11.848, *p* < 0.001). *Post hoc* analysis revealed significant differences between respondents who have smoked for less than 5 years and those with an experience of more than 8 years. For example, people who have smoked for 3–5 years differ significantly from those who have smoked for 8–10 years (mean difference = 1.703, *p* < 0.001) and from those who have smoked for more than 10 years (mean difference = 1.695, *p* < 0.001). Similarly, respondents with 0–3 years of experience differ significantly from those in the 8–10 years category (mean difference = 1.082, p = 0.001) and from those with over 10 years (mean difference = 1.074, *p* = 0.004). From these results, the data suggests that people with a shorter smoking duration tend to exhibit greater behavioral flexibility, being more open to changing the type of product (e.g., transitioning to electronic cigarettes) or quitting. In contrast, smokers with longer experience seem to exhibit increased behavioral stability, more frequently maintaining their traditional consumption habits.

#### 4.1.3 Relationship with holding a conventional cigarette

We noted that the perception of the role of holding a cigarette in the smoking experience varies significantly depending on the evolution of smoking behavior, as confirmed by the ANOVA analysis (*F* = 9.048, *p* < 0.001). *Post hoc* tests revealed significant differences between certain groups, especially between those who switched from conventional to electronic cigarettes and those who remained with traditional consumption. For example, respondents who no longer smoke (category 4) evaluate the importance of holding a cigarette significantly lower compared to those who still smoke conventionally (group 1; mean difference = 1.978, *p* < 0.001) or those who smoke both types (group 3; mean difference = 2.124, *p* < 0.001). Also, the scores of those who switched from conventional to electronic (group 2) are lower than those who continue to smoke conventionally, but the difference is only significant compared to group 4 (mean difference = 1.287, *p* = 0.020). Interpreting these results, it appears that quitting smoking or transitioning to modern alternatives is associated with a decrease in the importance of the physical ritual of smoking. In contrast, traditional smokers or those who combine both types of products seem to give greater meaning to the gesture of holding a cigarette, suggesting a stronger behavioral and symbolic attachment to this habit.

#### 4.1.4 Relationship with health

We found, based on the ANOVA results, a significant association between the perception of improved health status and the evolution of smoking behavior (*F* = 7.725, *p* < 0.001). *Post hoc* tests showed that people who continue to smoke conventionally (group 1) are significantly more skeptical about this change, compared to those who switched to electronic cigarettes (group 2) (mean difference = −1.89, *p* < 0.001) or those who started with electronic variants and continue with them (group 5) (difference = −1.33, *p* = 0.021). In our view, these results suggest that traditional smokers tend not to recognize the perceived benefits of the transition to less harmful alternatives, unlike those who directly experienced the change.

#### 4.1.5 Relationship with social acceptance

Based on the results obtained, we consider that acceptability in public spaces becomes a decisive argument in the orientation toward e-cigarettes and in maintaining this behavior and plays an important role for people who choose electronic cigarettes, either exclusively or in combination with conventional ones, compared to traditional smokers. ANOVA analysis confirmed significant differences between smoking evolution groups in relation to the reason for the social acceptability of electronic cigarettes (*F* = 8.046, *p* < 0.001). *Post hoc* tests indicate that people who switched from conventional to electronic smoking (group 2) and those who smoke both types (group 3) attribute a significantly higher score to this motivation compared to exclusive smokers of conventional cigarettes (group 1) (differences = +1.42 and +1.67; *p* < 0.01). In addition, people who started directly with electronic cigarettes and continue to use them (group 5) also consider social acceptability to be more important than group 1 (difference = +1.59; *p* = 0.017).

#### 4.1.6 Relationship with aesthetics

A one-way ANOVA indicated significant differences between the smoking evolution groups in the perceived aesthetic appearance of e-cigarettes (*p* < 0.001). Post hoc tests showed that exclusive e-cigarette smokers (group 2) and those who switched from conventional to electronic cigarettes (group 5) rated the aesthetics of e-cigarettes significantly higher than exclusive conventional smokers (group 1), with mean differences of +1.92 and +1.98 points, respectively (both p < 0.001). Respondents in group 4 (former smokers) assigned significantly lower aesthetic scores than groups 2 and 5, suggesting that positive aesthetic perceptions are more strongly associated with current e-cigarette users.

### 4.2 Sensory perceptions and patterns of cigarette use

The ANOVA analysis revealed a number of significant differences (*F* = 9.592, p < 0.001) between the smoking history groups regarding the perception of unpleasant taste in the mouth after smoking. Post hoc tests show that people who smoke both types of cigarettes (group 5) report significantly greater discomfort than all other groups, especially compared to: exclusively conventional smokers (group 1) → difference of −2.86, *p* < 0.001, those who switched from electronic to conventional (group 6) → difference of −3.15, *p* = 0.022, or former smokers (group 4) → difference of −2.91, *p* < 0.001. We believe that this situation reflects the fact that the combined use of conventional and electronic cigarettes is associated with increased post-smoking taste discomfort, possibly due to the interaction between the two types of consumption.

In addition to these aspects, gender appears to significantly influence the distribution of smoking behaviors. Women, who account for 70.8% of the sample, reported more frequent use of e-cigarettes, with 62 participants (23.2% of the total sample) using e-cigarettes, compared to 16 male participants (6.0% of the total sample). However, men were more likely than women to report using both conventional and electronic cigarettes, with 16 male respondents (6.0% of the total sample) versus 2 female respondents (0.7% of the total sample) indicating dual use.

The question regarding the type of cigarettes currently consumed shows a diversification of smoking habits. Thus, 29.2% of respondents declare that they smoke exclusively electronic cigarettes, 28.8% continue to smoke only conventional cigarettes, and 27.7% consume both types. Only 14.2% of participants no longer smoke at all. These data reflect an important variety in consumption behaviors and suggest distinct trends in preferences for tobacco products.

Regarding the initial influences on the decision to smoke, almost half of the respondents (49.1%) reported the group of friends as the main influencing factor, highlighting the role of peer context in smoking initiation. Also, a significant percentage (45.3%) claims that no one directly influenced them, which may indicate either a self-justification of the personal decision or an internalization of the decision to smoke. Only 3.4% were influenced by family, and the percentages for other sources are insignificant.

### 4.3 Attitudes and perceptions toward smoking and electronic cigarettes

Respondents’ attitudes and perceptions toward smoking and the use of electronic cigarettes highlight a series of psychosocial landmarks relevant to understanding consumption behavior.

A first dimension that we analyzed concerns the symbolic role of the cigarette in the act of smoking (Supplementary Appendix Table A4). Thus, a significant proportion of respondents (25.1%) declare that holding a cigarette “always” contributes to the smoking experience, while another 20.6% claim that it contributes “often.” Only 12.7% completely reject this idea, suggesting a ritualistic or identity function of the act of holding a cigarette, beyond the actual consumption of nicotine. The results highlight the fact that the variable “Holding a cigarette contributes to the experience and sensation of smoking” (Q11) presents significant and positive correlations with most of the items included in the analysis, which suggests a complex and symbolic role of this gesture within the framework of consumption behavior.

This indicator is also influenced by other perspectives, which amplify the experience of smoking. Using Pearson correlation analysis, we were able to explore the relationship between the perception of the gesture of holding a cigarette and various psychological and behavioral dimensions associated with smoking.

We identified the strongest association between Q11 and emotional and social motivations (Supplementary Appendix Table A5): smoking is perceived as a way to relax (Q20, *r* = 0.443, *p* < 0.01), reduce irritability (Q35, *r* = 0.426, *p* < 0.01), facilitate social interactions (Q36, *r* = 0.453, *p* < 0.01), as well as a coping mechanism in situations of emotional stress (e.g., Q41 – “When I am angry with someone, a cigarette helps me cope,” *r* = 0.367, *p* < 0.01). These correlations indicate that the gesture of holding a cigarette is not just an automatism, but acquires an affective and social function in the smoker’s life.

In parallel, we also observed significant correlations with variables reflecting awareness of addiction: Q25 – “Every cigarette maintains my addiction” (*r* = 0.267, *p* < 0.01), Q26 – “I will become more addicted if I continue smoking” (*r* = 0.267, *p* < 0.01), Q32 – “Cigarettes are gaining more and more control over me” (*r* = 0.222, *p* < 0.01), and Q23 – “The more I smoke, the harder it will be for me to quit” (*r* = 0.208, *p* < 0.01). Thus, we emphasize that although smoking is associated with momentary benefits, respondents also recognize the risk of escalating addiction over time.

At the same time, positive sensory perception of smoking (Q11) shows a small but significant positive correlation with the perception of pleasant taste while smoking (Q33: “When I smoke, the taste is pleasant”; *r* = 0.245, *p* < 0.01) and a moderate positive correlation with using smoking to fill time (Q34: “I smoke to pass the time”; *r* = 0.390, *p* < 0.01). In addition, a small but significant positive correlation was found between Q11 and the duration of tobacco consumption (Q7: “How long have you been smoking?”; *r* = 0.140, *p* < 0.05), suggesting that positive sensory perceptions may become more salient as smoking behavior becomes more chronic.

Overall, the data we analyzed indicate that holding a cigarette is closely linked to the affective, social, and cognitive dimensions of smoking, supporting the hypothesis that this gesture is not meaningless, but is an integral part of the smoker’s identity and the ritual of consumption.

Regarding the attractiveness of flavors as a motivation for using e-cigarettes, 39.3% of respondents “strongly agree” with the statement that the varied flavors constitute a reason for consumption, and 16.5% “agree.” This perception indicates an increased attraction for the sensory characteristics of the product, especially among young people. Furthermore, we applied a one-way ANOVA test for several relevant statements. The results show that the statement that “people smoke e-cigarettes because they have different flavors than classic ones” (Q12) presents a statistically significant difference depending on the duration of smoking [*F*(5,261) = 2.479, *p* = 0.032]. This value indicates that the perception of the distinct flavors of e-cigarettes is significantly influenced by the duration of smoking, which may suggest that the experience accumulated over time shapes sensory preferences or openness to alternatives. The perception of health status shows that 93% of respondents currently consider themselves healthy. Even in this context, among those who assessed their health status as ‘good’, 202 are active smokers. This optimistic self-assessment may reflect either denial of the risks or a tendency to downplay the negative effects of smoking. This indicator of flavor attractiveness can also be directly correlated with the following indicator Q13 (*r* = 0.198).

A more nuanced perception is found regarding the supposed effectiveness of electronic cigarettes in reducing health risks. Only 15% of respondents “totally agree” that switching to these products represents a healthier decision, while 45.3% express forms of disagreement, which suggests a moderate trust in the industry narrative regarding the “safety” of electronic alternatives.

At the same time, the social acceptability of electronic cigarettes emerges as an important motivational factor: 30.7% of participants state that they smoke these products because they can be used in spaces where conventional ones are prohibited, which reflects both a behavioral adaptation and a perception of “increased freedom” associated with vaping.

Another perspective we analyzed is that related to the aesthetics and costs associated with electronic cigarettes. Regarding the visual aspect, we find that 24.7% of respondents say that they are more attractive than conventional ones, while 36.6% disagree, suggesting a polarization of opinion. Regarding costs, only a quarter of participants (24.7%) consider that electronic smoking is cheaper, while a significant percentage remains neutral or skeptical (18% neutral; 35.5% disagree).

In terms of motivation to choose electronic cigarettes, curiosity appears as a frequently cited reason for initiating the use of them. Thus, the results of our analysis show that 25.5% of respondents chose to try them “out of curiosity,” and another 15.7% partially support this idea, indicating the exploratory and recreational nature of the behavior. It is important not to forget a hedonic dimension of smoking, which is confirmed by the item on taste pleasure. Approximately 26% “strongly agree” that taste contributes to the experience, and 17% “partially agree.” Only 8% completely disagree with this statement, suggesting a positive perception of the sensory characteristics associated with smoking, regardless of the type of product used.

### 4.4 Psychological and emotional motivations associated with smoking

The item (Supplementary Appendix Figure B1) “I’m not thinking of giving up smoking anytime soon” (Q19) provides a clear perspective on the intention to quit smoking among respondents and allows the assessment of the level of resistance to changing tobacco consumption behavior. The distribution of responses highlights the fact that 28.1% of participants declare that they completely agree with this statement, which indicates a strongly stabilized attitude in favor of continuing smoking. Together with those who selected “agree” (11.6%) and “slightly agree” (11.2%), it results that approximately 51% of respondents express some form of resistance to the idea of quitting smoking. On the other hand, only 19.5% completely disagree, and a cumulative percentage of 37.5% (including the responses “disagree” and “slightly disagree”) express intentions or attitudes closer to the possibility of quitting. Almost 12% of respondents chose the “neutral” answer, indicating an uncertain or ambivalent position.

Regarding smoking as a form of relaxation, item Q20 captures one of the most frequently cited psychological motivations for tobacco use: smoking as a strategy for emotional regulation and stress reduction. Analysis of the distribution of responses reveals strong support for this motivation. Almost 39% of respondents indicated that they “totally agree” with this statement, and if we also include the responses “agree” (14.6%) and “slightly agree” (15.7%), we obtain a total of approximately 69% of participants who recognize a relaxing role attributed to smoking. In contrast, only 18.7% of respondents expressed disagreement to varying degrees (from “totally disagree” to “slightly disagree”), which suggests that a relatively small proportion of the investigated population does not perceive smoking as being associated with relaxation. The remaining 12.4% remained neutral, possibly indicating an ambivalent or context-dependent relationship between smoking and relaxation.

In terms of using cigarettes as a strategy to combat boredom (Q34) or lack of activity, the analyzed results show that a significant proportion of respondents (36.6%) are in total agreement, and another 26.1% are in the agreement zone (“agree” - 12.1%; “slightly agree” - 14.0%), totaling over 62% of participants who recognize smoking as a form of occupation in the absence of other activities. On the other hand, 27.9% express disagreement to varying degrees, while 9.4% adopt a neutral position. We thus highlight a passive-compensatory function of smoking, in which the act of smoking becomes an automatic behavior, integrated into moments of inactivity or daily monotony.

Furthermore, we also tried to analyze the role of cigarettes in regulating negative emotions (Q35), especially irritability — a common state in everyday life and an important trigger for smoking behavior. The results are quite telling: 44.9% of respondents selected “strongly agree,” and another 27.6% are in the “agree” (15.5%) and “slightly agree” (12.1%) categories. In total, we found that almost 73% of participants attribute a clear calming function to cigarettes in situations of irritation. The number of those who disagree is relatively low (17.7%), and 9.8% remain neutral, indicating a potential ambivalence. This shows that for a majority of smokers included in the sample, smoking is not only a physiological or social act, but also a deeply internalized emotional regulation strategy.

Items Q36, Q37, Q38 highlight the complexity of motivations that support smoking behavior, from social integration and sensory pleasure, to the regulation of negative emotions.

The psychosocial dimension of smoking (Q36) investigates whether cigarettes function as a facilitator of social interaction and a reducer of social anxiety. The distribution of responses suggests a relatively balanced split, but with a slight positive trend: 29.8% of respondents selected “strongly agree,” and another 19.6% agree to a lesser extent (“agree” and “slightly agree”). In total, almost 50% of respondents express agreement, which suggests a relational function of smoking, through which it is perceived as a tool of social comfort. However, 32.8% express forms of disagreement, and 17.7% adopt a neutral position, which may indicate an attitudinal division: for some, smoking is integrated into social rituals, while for others it has no interpersonal significance. These results highlight the fact that, in some contexts, the cigarette becomes an “intermediate object” in social relationships, reducing tension and increasing the sense of belonging.

In terms of the sensory-hedonic dimension of smoking (Q37), through which the gesture becomes a source of tactile and gustatory pleasure, the results indicate a moderate-high degree of sensory attachment: 23% declared “strongly agree,” and another 24.5% responded positively (“agree” + “slightly agree”), totaling almost 48% of responses reflecting a positive association between smoking and the physical sensation experienced. At the same time, approximately 35% of respondents express varying degrees of disagreement, and 18.5% adopt a neutral position. This polarization may reflect differences between passionate smokers and those who smoke more out of habit, stress or social pressure. Item (Q38) focuses on the function of negative emotional regulation, assessing the extent to which smoking is used as a mechanism for managing anger. Almost 32.5% of the participants selected “strongly agree,” and another 27.1% expressed moderate agreement (“agree” + “slightly agree”), totaling almost 60% of favorable responses. Thus, for a majority of respondents, the cigarette is perceived as an effective tool for relieving tension in angry situations. At the same time, 26% disagree to varying degrees, and 14.3% are neutral. Although part of the sample does not recognize this role of smoking, the consistent presence of agreement indicates a strong internalization of smoking as an emotional strategy.

A comparative analysis of items Q39, Q40 and Q41 clearly reveals the emotional self-regulation function attributed to smoking among respondents. In the case of item Q39 (“Cigarettes help me deal with anxiety or worry”), a significant proportion – 37.5% of respondents – declare that they completely agree with the statement, and another 26.5% express different degrees of agreement (agree or slightly agree). Therefore, over 64% of respondents perceive smoking as a tool for managing anxiety, which indicates a complex psycho-emotional addiction, in which the cigarette becomes a means of calming down in the face of uncertainty or daily stress. Only 24.6% reject this association, signaling a minority that does not link tobacco consumption to anxiety. This trend is also reinforced by the data from Q40 (“Cigarettes help me reduce or handle tension”), where 35.6% of respondents choose the option strongly agree, and another 27.7% fall into the spectrum of agreement. Cumulatively, over 63% of participants recognize smoking as a method of reducing tension. Thus, the data confirm a clear coping function, with smoking used as a release valve for emotional tension, possibly in the absence of adaptive alternatives (e.g., physical exercise, communication, cognitive self-regulation).

Through item Q41 (“When I’m upset with someone, a cigarette helps me cope”) we add an interpersonal nuance to the overall picture. Approximately 33.7% declare strong agreement, and another 27.3% are on the positive spectrum. In total, 61% of respondents use smoking as a means of calming down in conflict situations, which suggests a form of external emotional regulation, in which reactions to frustration or conflict are not managed through mature behavioral mechanisms, but by resorting to nicotine.

In analyzing the psychological and emotional motivations that support smoking behavior (Supplementary Appendix Figure B2), the data help us to outline a complex model, in which smoking is perceived not only as a habit, but also as an adaptive mechanism in the face of emotional and social discomfort. The significant and consistent correlations between the items are useful in outlining a well-defined profile of the smoker who uses cigarettes as a compensatory strategy in a wide range of stressful or tense situations. We significantly correlate item Q19 (“I’m not thinking of giving up smoking anytime soon”) with all the other analyzed dimensions, especially with Q20 (“I smoke to relax,” *r* = 0.502), suggesting that the intention to quit is weak among those who perceive smoking as a source of relaxation. This association is reinforced by a dense network of correlations revolving around emotional variables such as Q35 (“If I’m irritable...,” *r* = 0.653), Q38 (“...deal with anger,” *r* = 0.586) and Q39 (“...deal with anxiety or worry,” *r* = 0.562). Therefore, smoking cessation is not an end in itself, but a desired effect in the face of psychological discomfort.

At the same time, we noted the existence of a coherent affective core, formed by items Q38–Q41, whose correlations exceed the threshold of 0.77 (e.g., Q38–Q39: *r* = 0.803; Q39–Q40: *r* = 0.832). These values indicate that, for a significant part of the respondents, smoking becomes a universal tool for managing negative emotions, such as anger, anxiety, tension and interpersonal conflicts. We identify the presence of the social dimension, but with a somewhat lower intensity. We observe through item Q36 (“I feel more at ease with others if I have a cigarette”) moderate to high correlations with the affective variables (e.g., with Q41, *r* = 0.611), suggesting that smoking plays a role in facilitating social relationships, especially in tense contexts. In addition, the sensory dimension (Q37 – “I enjoy feeling the smoke...”) is significantly correlated with all other dimensions, but with more moderate intensities (r between 0.39 and 0.53), indicating a subordinate contribution to emotional motivations.

### 4.5 Smokers patterns

Based on the K-Means Cluster analysis, we conducted a broad set of demographic, attitudinal and behavioral variables regarding smoking, and identified six distinct profiles of smokers. Each profile was defined by the average scores of respondents on items measuring motivations and attitudes toward conventional and electronic cigarettes.

#### 4.5.1 Low-involved and cognitively detached smokers

This group, consisting of 21 respondents, presents a profile marked by very low scores on all motivational, cognitive and emotional dimensions of smoking. Items Q19–Q41, which measure attitudes regarding addiction, relaxation or the social function of smoking, were evaluated with scores between 1 and 2 on average. We observe that these individuals show minimal behavioral involvement, being most likely occasional smokers or in the early stages of consumption.

#### 4.5.2 Functional, moderate and conscious smokers

The second cluster, consisting of 42 respondents, brings together individuals who smoke regularly, but the main motivation seems to be of a functional and situational nature, not addictive. Moderate scores (between 3 and 5) on items such as Q20 (relaxation), Q34 (boredom), Q35 (irritability) indicate a use of smoking similar to a contextual stress management tool, without a deep emotional anchoring.

#### 4.5.3 Addicted but reflective smokers

Cluster 3 (*N* = 38) offers a paradoxical profile: respondents register high scores on items indicating a strong physical and emotional dependence (Q19, Q20, Q35–Q41). Thus, we find that individuals in this category face cognitive dissonance: although they clearly recognize the negative health consequences of smoking, they continue the behavior due to an intense attachment or a compensatory function.

#### 4.5.4 Dual and moderately involved users

The fourth group, with 29 members, seems to bring together individuals who combine conventional and electronic smoking, without developing a marked addiction. Moderate scores on the hedonistic (Q33–Q37) and functional (Q34–Q35) variables suggest an adaptive consumption to social contexts, but with a low level of emotional and physical involvement toward the environment. However, the analyzed data showed us that the aesthetic appeal and accessibility of electronic cigarettes (Q14–Q18) are significant.

#### 4.5.5 Hedonistic smokers, focused on pleasure

Within this cluster (*N* = 36), we identified a motivational profile characterized by a strong emphasis on the value of sensory pleasure, aesthetics, and facilitating social interaction. Items such as Q33 (taste pleasure), Q16 (aesthetic attractiveness) and Q36 (ease of relating) register significantly high scores, suggesting a predominantly hedonic behavioral orientation, rather than one marked by physiological dependence. Such an attitudinal pattern is likely to be encountered with greater frequency among young adults, especially those for whom smoking functions as an element of identity or as a means of consolidating social status and group integration. In this context, we can state that intervention strategies are recommended to be built on a non-moralizing framework, avoiding a prescriptive or sanctioning tone, and oriented toward highlighting the unfavorable aesthetic and social consequences of smoking in the long term, aspects that may have a stronger resonance among this segment of the population.

#### 4.5.6 Emotional smokers with high addiction

The largest group (*N* = 88) is characterized by very high levels of emotional and physiological dependence. Scores on items Q35–Q41 reach maximum values, suggesting a constant use of smoking as an emotional coping mechanism (e.g., managing anger, anxiety, tension, or interpersonal conflicts).

This cluster analysis was useful because we identified six distinct psychobehavioral patterns, each with its own motivational and cognitive nuances. The segmentation we presented provides us with valuable tools for personalizing public health interventions and developing differentiated messages in anti-smoking campaigns.

## 5 Discussion

By relating empirical data to the formulated theoretical hypotheses, the investigative approach aimed not only examine these hypotheses in terms of observed associations but also to outline some explanatory directions regarding the dynamics of smoking behavior among students.

In testing hypothesis H1, we started from the premise that students who started smoking with conventional cigarettes, which presents a higher probability of maintaining the consumption of this type of product in the long term, compared to those who started with electronic cigarettes. The data we obtained from the statistical analysis are consistent with this hypothesis. The Chi-square test of independence [χ^2^(3) = 20.288, *p* < 0.001], along with the values of the Phi and Cramér’s *V* coefficients (both = 0.276), highlight a significant and moderate intensity association between the type of cigarette used at the beginning and current behavior. In addition, the Kendall’s tau-b coefficient = 0.150 (*p* = 0.009) further supports this relationship also from the perspective of an ordering of the variables, thus strengthening the interpretation of the data. Specifically, among those who started to consume conventional cigarettes (80.1% of the sample), a substantial proportion continues to smoke them exclusively (27%) or in combination with electronic cigarettes (27.7%). This fidelity to the debut product may suggest a psychobehavioral anchoring in traditional habits, potentially influenced both by accessibility and by the ritualistic dimension of conventional smoking (e.g., the gesture of holding the cigarette, the smell, the sensation in the oral cavity). Encapsulated are the ritualistic and symbolic qualities regarding smoking (Pelters and Galanti, 2023). For example, many smokers developed rituals, and used specific artifacts and micro-behaviors to construct artisanal cigarettes and imbue RYO (roll-your-own) with positive attributes (Hoek et al., 2016). In some countries in Asia, such as India, both smoking and nonsmoking forms of tobacco are deeply ingrained in cultural and regional customs (More et al., 2025). Ultimately, previous studies suggest that self-control can limit the desire of students to reduce or stop smoking (Mujidin et al., 2024). Scientists are still learning about the health effects of e-cigarette, but the available science shows they contain harmful and potentially harmful ingredients (Salama et al., 2022).

Hypothesis H2 states that a positive perception of the aesthetics and aroma of e-cigarettes is associated with an increased likelihood of transitioning from conventional to e-cigarette smoking. A coherent set of relevant statistical results is in line with this hypothesis. ANOVA analysis, complemented by *post hoc* Tukey HSD tests, indicates the presence of significant differences between smoking evolution groups reported on aesthetic perceptions. Thus, participants who made the transition from conventional to e-cigarettes, as well as those who continued to use e-cigarettes exclusively, evaluated their aesthetic appearance in a significantly more favorable manner than exclusive smokers of conventional products (differences of +1.92 and +1.98, *p* < 0.001). In addition to the aesthetic component, the results also suggest the important role of flavors in motivating the choice of electronic products: 39.3% express “totally agree,” and another 16.5% “agree” that the variety of flavors is an important factor associated with the adoption of this type of product. The unifactorial ANOVA analysis applied to statement Q12 (“People smoke electronic cigarettes because they have different flavors”) revealed a statistical significance depending on the duration of smoking behavior [*F*(5,261) = 2.479, *p* = 0.032], suggesting the presence of a relationship between the length of tobacco product consumption and openness to the sensory characteristics of electronic cigarettes. This finding supports the idea that the adoption of electronic smoking is not explained exclusively by health considerations or the intention to reduce risks, but is also associated with a relevant aesthetic dimension, which may contribute to a distinct and attractive consumption experience, especially for the young audience, hence why it is found out and accepted that devices have gained popularity among youth and young adults due to their appealing flavors and the perception that they are less harmful than traditional tobacco cigarettes (Bieganek et al., 2024).

This societal aspect has been called the cigarette epidemic (Östergren, 2022). In this regard, smoking reduction campaigns should counterbalance the discourse promoted by the tobacco industry regarding the aesthetic or style advantages of alternative products, integrating a series of conclusive and credible messages that highlight the potential long-term harmful effects, even of electronic cigarettes. An example is smoking cessation interventions (Onwuzo et al., 2024) and smoke cessation therapies (Marshall et al., 2023). E-cigarettes can aid smoking cessation and reduce carcinogen exposure (Andrayas et al., 2024).

Hypothesis H3 claims that students who smoke to manage negative emotions will show a reduced intention to quit smoking and will present a specific behavioral pattern. This hypothesis is supported by a dense network of significant correlations linking smoking to emotional and social functions. Thus, item Q19 (“I do not think about quitting smoking”) is positively and significantly correlated with items such as: Q20 (“I smoke for relaxation,” *r* = 0.502), Q35 (“to reduce irritability,” *r* = 0.653), Q38 (“to deal with anger,” *r* = 0.586), Q39 (“to manage anxiety,” *r* = 0.562), Q41 (“when I am angry with someone...,” *r* = 0.367). Cluster analysis, in turn, reinforces these findings. The largest group identified (*N* = 88) was that of “highly dependent emotional smokers,” whose structure is characterized by maximum scores on all variables associated with the regulation of negative emotions. Smoking is, in this case, internalized not as a vice necessarily, but rather catalogued as an adaptive mechanism of affective coping; as such, coping with stress has been associated with reduced cravings among smokers attempting to quit, coping with stress reduces craving through negative effect, but only for a limited timeframe (Kumar et al., 2022).

From a theoretical perspective, the data we obtained can be related to the emotional dependence model and the concepts of “deficit self-regulation,” dependent behavior can be represented by actions, signs, and symptoms (Santos and Diniz, 2024) and Emotional Dependency is a disorder characterized by addictive behaviors (i.e., most commonly romantic relationships) (Bution and Muglia-Wechsler, 2016). In the absence of effective adaptive strategies (e.g., assertive communication, sports, self-reflection), individuals may resort to cigarette consumption as a perceived quick and effective form of calming down. However, this behavior of “self-medication” with nicotine has been described in the literature as problematic, as it may maintain the cycle of addiction and may diminish the motivation to quit. Consequently, the results raise an important question regarding the effectiveness of messages focused exclusively on physical or financial risks. We can emphasize that for these individuals, smoking is not a rational choice, but rather an emotional refuge. Given the cross-sectional design of the study, these patterns should be interpreted as associations rather than causal relationships, and the proposed explanatory mechanisms remain tentative.

Taken together, our findings are consistent with the view that smoking is not a singular or uniform act, but a complex phenomenon, with a social, psychological, and sensory substratum. Smokers’ behavior appears to be shaped by a complex interaction between onset factors (type of debut cigarette), sensory perceptions (flavors, aesthetics), as well as deep affective motivations (relaxation, anger management, social belonging). Interestingly, it is still unknown whether a cigarette’s relative reinforcing efficacy can be predicted by these perceptions and whether this relationship may vary due to constituents known to alter those perceptions (Karelitz and Perkins, 2021). An explanatory study shows that the urge to smoke was indirectly affected by emotion dysregulation and negative affect via positive metacognitions about smoking, while nicotine dependence was indirectly affected by emotion dysregulation and negative affect through negative metacognitions about smoking (Poormahdy et al., 2022). At the same time, the use of cluster analysis allowed us to outline distinct behavioral typologies, which could be used to establish differentiated intervention strategies, adapted to the profile of each category of smokers: from hedonic or functional smokers, to those individuals who present a deep emotional dependence.

## 6 Conclusion

The smoking behavior among students analyzed in this research highlights a complex reality, shaped by a network of emotional, symbolic, and social factors. For a significant proportion of respondents, smoking remains a recurring practice and is justified by a varied palette of personal and interpersonal motivations. Beyond the simple satisfaction of a need, the act of smoking is often internalized as a mechanism for emotional regulation, being invested with the role of mediating negative affective states such as tension, anger, or anxiety. In many situations, the cigarette is invested with an affective function and ends up being viewed as an instrument of emotional control in moments of psychological discomfort. Also, the aesthetic, sensory and social dimension of smoking — especially in the case of electronic cigarettes — contributes to the perpetuation and normalization of the behavior. Attributes such as pleasant appearance, variety of flavors, acceptability in public spaces, and modern perception of electronic products form a favorable context for continued consumption, even in the absence of intense physiological dependence.

The transition between different forms of consumption (from conventional to electronic, or vice versa) is a frequent phenomenon and often determined by circumstantial factors such as social pressure, curiosity or the desire to reduce the visible effects of traditional smoking. These behaviors reflect a fluidity of choices and an increased adaptability of young consumers to new forms of smoking, but without signaling a real detachment from the product itself. At the same time, the act of holding a cigarette, regardless of its content, is symbolically invested with a role of identity, belonging and social comfort. The findings of this analysis indicate the need to reconfigure public health strategies regarding the consumption of tobacco products, emphasizing the complexity of motivations and behavioral patterns of young users.

In this regard, it is necessary to develop and implement integrated, multidimensional policies, which include the following directions:

1. Early psychosocial education – Introduction of educational modules for emotional health, stress management, and informed decision-making into the school curriculum, which would provide young people with non-addictive tools for emotional regulation.
2. Regulation of commercial communication – It is necessary to tighten the legislative framework that regulates the promotion of electronic cigarettes, especially in digital environments frequented by young people, and to implicitly prohibit messages that associate these products with freedom, attractiveness or modernity.
3. Differentiated communication campaigns – Building prevention and information campaigns adapted to the psychological profile of different types of smokers (e.g., hedonistic smokers vs. emotional smokers), which would go beyond moralizing approaches and use clear and relevant messages, based on understanding the daily realities of the target audience.
4. Access to psychological support services – Establishing counseling centers accessible to young people in educational institutions and universities, where smoking can be approached not only as a physical addiction, but similar as a symptom generated by an unaddressed emotional need.

Our research also presents a series of methodological limitations that must be assumed with caution in interpreting the results. First, the use of a non-probabilistic sampling along with the overweight of female respondents may affect the representativeness of the results and implicitly the generalizability of the conclusions. Second, the use of self-reporting may introduce subjective errors or social conformity bias. Also, the absence of a qualitative component limits the deep understanding of the motivations and experiences associated with smoking. Finally, the cross-sectional nature of the study prevents the formulation of firm causal conclusions.

## Data Availability

The original contributions presented in the study are included in the article/Supplementary material, further inquiries can be directed to the corresponding author/s.
