# Supplementary material for: Multidimensional analysis of conventional and electronic cigarette consumption among students
Source: Front Psychol. 2026 Jan 30;16:1724372. doi: 10.3389/fpsyg.2025.1724372 (PMC12903915; doi:10.3389/fpsyg.2025.1724372)
Supplement: Supplementary file 1 [file Supplementary_file_1.docx]

**Appendix A**

**Table A1. Themes and variables analyzed**

|  | Variable name | Variable code |
| --- | --- | --- |
| Socio-demographical data | Country of origin | Q0 |
|  | Gender | Q1 |
|  | Residential environment | Q2 |
|  | Age category | Q3 |
|  | Educational level | Q4 |
|  | Perceived health status | Q5 |
| Smoking behavior | Smoker status | Q6 |
|  | Duration of smoking | Q7 |
|  | Type of cigarette used at first | Q8 |
|  | Type of cigarette used currently | Q9 |
|  | Who influenced the first decision to smoke | Q10 |
| Perceptions and attitudes | The pleasure of holding a cigarette | Q11 |
|  | Different flavors of e-cigarettes | Q12 |
|  | Switching to e-cigarettes improves health | Q13 |
|  | E-cigarettes are accepted in more places | Q14 |
|  | There are better ways to quit than e-cigarettes | Q15 |
|  | The aesthetics of e-cigarettes | Q16 |
|  | E-cigarettes are cheaper | Q17 |
|  | The curiosity to try e-cigarettes | Q18 |
|  | The pleasant taste of smoke | Q33 |
| Psychological and emotional motivations | I don't plan to quit smoking | Q19 |
|  | I smoke to relax | Q20 |
|  | I smoke when I have nothing to do | Q34 |
|  | Smoking calms me down when I’m irritated | Q35 |
|  | I feel more socially comfortable when I smoke | Q36 |
|  | I like the feeling of smoke in my mouth/throat | Q37 |
|  | Smoking helps me with anger | Q38 |
|  | Smoking helps me with anxiety or worry | Q39 |
|  | Smoking helps me manage tension | Q40 |
|  | Smoking helps me when I’m angry with someone | Q41 |

**Source: Database processing**

| **Table A2. 8. Which type of cigarette did you start smoking at first? * 9. What type of cigarettes do you currently smoke? Crosstabulation** | | | | | | |
| --- | --- | --- | --- | --- | --- | --- |
| Count | | | | | | |
|  | | 9. What type of cigarettes do you currently smoke? | | | | Total |
|  |  | Both | Conventional | Electronic | Neither |  |
| 8. Which type of cigarette did you start smoking at first? | Conventional | 62 | 72 | 51 | 29 | 214 |
|  | Electronic | 12 | 5 | 27 | 9 | 53 |
| Total | | 74 | 77 | 78 | 38 | 267 |

**Source: Database processing**

| **Table A3. Evolution of smoking from conventional to electronic** | | | | | |
| --- | --- | --- | --- | --- | --- |
|  | | Frequency | Percent | Valid Percent | Cumulative Percent |
|  | 1. From conventional → conventional (Q8=1 and Q9=2) | 72 | 27,0 | 27,0 | 27,0 |
|  | 2. From conventional → electronic (Q8=1 and Q9=3) | 51 | 19,1 | 19,1 | 46,1 |
|  | 3. Smokes both types now (Q9=1) | 74 | 27,7 | 27,7 | 73,8 |
|  | 4. No longer smokes (Q9=4) | 38 | 14,2 | 14,2 | 88,0 |
|  | 5. From electronic → electronic (Q8=2 and Q9=3) | 27 | 10,1 | 10,1 | 98,1 |
|  | 6. From electronic → conventional (Q8=2 and Q9=2) | 5 | 1,9 | 1,9 | 100,0 |
|  | Total | 267 | 100,0 | 100,0 |  |

**Source: Database processing**

| **Tabel A4. 11. Holding a cigarette contributes to the experience and feeling of smoking (1 being the lowest, 7 being the highest):** | | | | | |
| --- | --- | --- | --- | --- | --- |
|  | | Frequency | Percent | Valid Percent | Cumulative Percent |
| Valid | Never | 34 | 12,7 | 12,7 | 12,7 |
|  | Rarely | 18 | 6,7 | 6,7 | 19,5 |
|  | Occassionally | 25 | 9,4 | 9,4 | 28,8 |
|  | Sometimes | 40 | 15,0 | 15,0 | 43,8 |
|  | Often | 55 | 20,6 | 20,6 | 64,4 |
|  | Very often | 28 | 10,5 | 10,5 | 74,9 |
|  | Always | 67 | 25,1 | 25,1 | 100,0 |
|  | Total | 267 | 100,0 | 100,0 |  |

**Source: Database processing**

**Table A5. What factors contribute to the symbolic role of the cigarette in the act of smoking**

|  | Q11 | Q7 | Q19 | Q20 | Q23 | Q25 | Q26 | Q32 | Q33 | Q34 | Q35 | Q36 | Q37 | Q38 | Q39 | Q40 | Q41 |
| --- | --- | --- | --- | --- | --- | --- | --- | --- | --- | --- | --- | --- | --- | --- | --- | --- | --- |
| Q11 | 1 | 0,14 | 0,236 | 0,443 | 0,208 | 0,267 | 0,267 | 0,222 | 0,245 | 0,39 | 0,426 | 0,453 | 0,329 | 0,34 | 0,338 | 0,375 | 0,367 |
| Q7 |  | 1 | 0,381 | 0,264 | ,000 | ,020 | ,070 | ,052 | ,206^**^ | ,135^*^ | ,242^**^ | ,080 | ,112 | ,221^**^ | ,194^**^ | ,159^**^ | ,152^*^ |
| Q19 |  |  | 1 | ,502^**^ | ,119 | ,125^*^ | ,004 | -,106 | ,396^**^ | ,276^**^ | ,382^**^ | ,231^**^ | ,320^**^ | ,296^**^ | ,263^**^ | ,275^**^ | ,253^**^ |
| Q20 |  |  |  | 1 | ,223^**^ | ,256^**^ | ,175^**^ | ,066 | ,431^**^ | ,440^**^ | ,653^**^ | ,517^**^ | ,394^**^ | ,586^**^ | ,562^**^ | ,567^**^ | ,520^**^ |
| Q23 |  |  |  |  | 1 | ,673^**^ | ,619^**^ | ,436^**^ | ,236^**^ | ,259^**^ | ,243^**^ | ,333^**^ | ,161^**^ | ,267^**^ | ,270^**^ | ,269^**^ | ,279^**^ |
| Q25 |  |  |  |  |  | 1 | ,669^**^ | ,458^**^ | ,282^**^ | ,304^**^ | ,303^**^ | ,359^**^ | ,283^**^ | ,306^**^ | ,303^**^ | ,328^**^ | ,315^**^ |
| Q26 |  |  |  |  |  |  | 1 | ,541^**^ | ,178^**^ | ,232^**^ | ,259^**^ | ,316^**^ | 0,11 | ,267^**^ | ,263^**^ | ,309^**^ | ,310^**^ |
| Q32 |  |  |  |  |  |  |  | 1 | ,155^*^ | ,254^**^ | ,244^**^ | ,291^**^ | ,149^*^ | ,222^**^ | ,243^**^ | ,258^**^ | ,225^**^ |
| Q33 |  |  |  |  |  |  |  |  | 1 | ,367^**^ | ,476^**^ | ,386^**^ | ,499^**^ | ,422^**^ | ,441^**^ | ,410^**^ | ,401^**^ |
| Q34 |  |  |  |  |  |  |  |  |  | 1 | ,562^**^ | ,540^**^ | ,399^**^ | ,499^**^ | ,493^**^ | ,443^**^ | ,483^**^ |
| Q35 |  |  |  |  |  |  |  |  |  |  | 1 | ,593^**^ | ,478^**^ | ,723^**^ | ,692^**^ | ,670^**^ | ,664^**^ |
| Q36 |  |  |  |  |  |  |  |  |  |  |  | 1 | ,441^**^ | ,516^**^ | ,566^**^ | ,551^**^ | ,611^**^ |
| Q37 |  |  |  |  |  |  |  |  |  |  |  |  | 1 | ,533^**^ | ,473^**^ | ,487^**^ | ,435^**^ |
| Q38 |  |  |  |  |  |  |  |  |  |  |  |  |  | 1 | ,803^**^ | ,777^**^ | ,773^**^ |
| Q39 |  |  |  |  |  |  |  |  |  |  |  |  |  |  | 1 | ,832^**^ | ,795^**^ |
| Q40 |  |  |  |  |  |  |  |  |  |  |  |  |  |  |  | 1 | ,778^**^ |
| Q41 |  |  |  |  |  |  |  |  |  |  |  |  |  |  |  |  | 1 |
| *. Correlation is significant at the 0.05 level (2-tailed). | | | | | | | | | | | | | | | | | |
| **. Correlation is significant at the 0.01 level (2-tailed). | | | | | | | | | | | | | | | | | |

**Source: Database processing**

**Appendix B**

**Fig B1. Perception in relation to psychological and emotional motivations associated with smoking
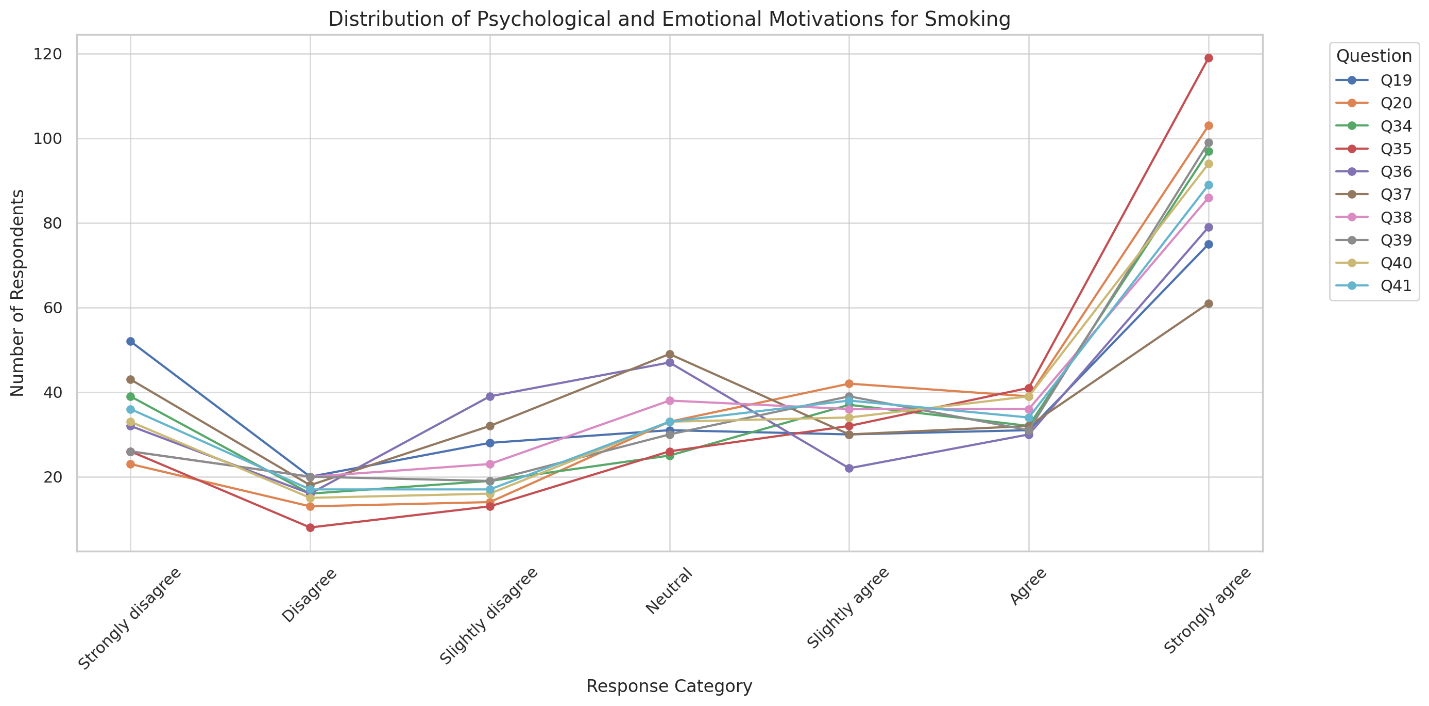
**

**Source: Database processing**

**Fig B2. Matrix of correlations regarding psychological and emotional motivations associated with smoking**

**Source: Database processing**
